# Biochemical reconstitution of UV-induced mutational processes

**DOI:** 10.1093/nar/gkz335

**Published:** 2019-05-04

**Authors:** Tomohiko Sugiyama, Yizhang Chen

**Affiliations:** Department of Biological Sciences, Ohio University, Athens, OH 45701, USA

## Abstract

We reconstituted two biochemical processes that may contribute to UV-induced mutagenesis *in vitro* and analysed the mutational profiles in the products. One process is translesion synthesis (TLS) by DNA polymerases (Pol) δ, η and ζ, which creates C>T transitions at pyrimidine dimers by incorporating two dAMPs opposite of the dimers. The other process involves spontaneous deamination of cytosine, producing uracil in pyrimidine dimers, followed by monomerization of the dimers by secondary UV irradiation, and DNA synthesis by Pol δ. The mutational spectrum resulting from deamination without translesion synthesis is similar to a mutational signature found in melanomas, suggesting that cytosine deamination encountered by the replicative polymerase has a prominent role in melanoma development. However, CC>TT dinucleotide substitution, which is also commonly observed in melanomas, was produced almost exclusively by TLS. We propose that both TLS-dependent and deamination-dependent mutational processes are likely involved in UV-induced melanoma development.

## INTRODUCTION

A major cause of skin cancer, ultraviolet light (UV)-induced DNA damage has been studied in great detail ((1,2) for reviews). The major DNA photoproducts produced by UV radiation are dimers of two adjacent pyrimidines (pyrimidine dimers) that include cyclobutane pyrimidine dimers (CPD), (6-4) photoproducts and their Dewar isomers. While most of these photoproducts are repaired by nucleotide excision repair (NER) in cells, unrepaired damage may lead to DNA mutation. UV-induced mutations occur predominantly at GC-pairs in yeast and humans (3,4). Large-scale cancer genome analysis (5,6) revealed a distinct pattern of base substitution (Signature 7) that is predominant in UV-induced melanoma. More than 90% of mutations in Signature 7 are CG-pair to TA-pair transitions (C>T transitions) in contiguous pyrimidine residues (5), supporting that the majority of the mutations occur at the pyrimidine dimers.

One well-accepted model of UV-induced mutagenesis involves spontaneous deamination of cytosine (C) residues in pyrimidine dimers (7,8). Photochemical studies observed C deamination in CC, CT and TC photodimers (9–14) where deamination in CPDs is remarkably faster (*t*_1/2_ = 2–20 hours, depending on the sequence and conditions) than spontaneous deamination of monomeric cytosine residues (*t*_1/2_ = more than 200 years (15)). The deamination converts a C residue to a uracil (U) in CPD, which can be converted to thymine (T) after two rounds of DNA replication (16,17). In addition, >80% of C residues in CpG sequences in the human genome are modified to 5-methylcytosine (5mC) (18), deamination of which directly produces T residues.

Translesion synthesis (TLS) is a mechanism in which specialized DNA polymerases (TLS polymerases) synthesize over DNA damage (19). Ample evidence supports that Pol η is a key enzyme in TLS over UV-induced DNA damage. Loss of human Pol η function is seen in the xeroderma pigmentosum variant (20,21), which had an elevated frequency of UV-induced mutations, mainly C>A transversion (22,23) but also C>T transition at TpC and CpC sites (24,25). Consistent with its suppressive role in mutagenesis, purified Pol η can mediate an error-free TLS by inserting two dAMPs opposite a TT dimer (26–30). While TLS may increase the chance of cell survival without repairing DNA damage, it may also increase mutation frequency. Although Pol η mediates error-free TLS over TT dimers, analyses to obtain exact fidelity of Pol η during TLS over C-containing pyrimidine dimers is not conclusive, largely because of the instability of the C moiety in dimers.

The TLS activity of Pol η is facilitated by Pol ζ that efficiently extends a mismatched 3′ end (31,32). In addition to Pol η, humans have at least three more conserved Y-family DNA polymerases; Pol ι, Pol κ and Rev1 (33–35). Biochemical studies have shown that Pol ι and Pol κ are error-prone DNA polymerases and have TLS activity *in vitro* (16,31,36–38). Several groups have reported that Pol ι is involved in the UV-induced mutagenic TLS at least in the absence of Pol η (39–42). Rev1 has limited polymerase activity that incorporates only dCMP into DNA (43). Rev1 also organizes TLS polymerases through protein-protein interaction with Pol ζ, Pol η and the PCNA sliding clamp (44–47). Despite these investigations, the exact roles of these polymerases in UV-induced mutagenesis have not been elucidated.

To obtain insight into the aetiology of skin cancer-causing mutations, we reconstituted *in vitro* UV-induced mutagenesis using purified proteins and model DNA substrates. To quantify the base substitution frequency, the DNA products were directly analysed by next generation sequencing (NGS)-based method (48).

## MATERIALS AND METHODS

### DNA

Sequences of all synthetic DNAs used in this study are shown in Supplementary Table S1. The structures of single-stranded DNA (ssDNA) templates (template A to G) are also shown in Supplementary Figure S2A. The double-stranded DNA (dsDNA) templates were created by annealing ssDNA templates with their complementary oligos (‘Top strand’ for each template, see Supplementary Table S1 and Figure 4A).

### CpG methyltransferase treatment

When indicated, dsDNA templates (2.0 μM) were treated with CpG methyltransferase (M.*Sss*I, New England Biolabs) as instructed by the manufacturer. After the reaction, methylation was confirmed by resistance to restriction digestion by *Pvu*I and *Sal*I on template A and D, respectively (Supplementary Figure S6).

### UV-irradiation

Twenty microliters of 200 nM DNA in PBS buffer (8.06 mM Na_2_HPO_4_, 1.47 mM KH_2_PO_4_, 2.67 mM KCl and 138 mM NaCl, pH 7.3) was irradiated in a well of 60-well HLA Terasaki microtiter plate with the desired dose of UV. A UV-Stratalinker (Stratagene) was used as the source of UVC (254 nm) radiation, relying on its internal UV-meter. A UVP Benchtop variable transilluminator with USHIO G8T5E bulbs was used for UVB (peak at 302 nm) radiation. The intensity of UVB was measured with a Sper Scientific UVA/B light meter. Typically, 1.0 kJ/m^2^ radiation of UVC and UVB was obtained by 22.5 and 23.5 s of exposure, respectively. When indicated, irradiated DNA in PBS buffer was incubated at 37°C to facilitate deamination, and then irradiated again in the same buffer to induce photoreversion. To minimize undesired spontaneous deamination, irradiated DNA samples were immediately stored at –80°C in the dark and thawed immediately before the primer extension reaction.

### Proteins

yPol δ, yPol ϵ (catalytic subunit), yPol η and yPol ζ were purified as described (48,49). Human cDNA of *POLH* (isoform 1, encoding 713 amino acids), *POLI* (isotype a (short), encoding 715 amino acids), *POLK* (isotype 1: encoding 870 amino acids), and the yeast *REV1* open reading frame were amplified by PCR and cloned into pET21a to fuse their C-termini with a His6 tag. After confirming the DNA sequences, the plasmids were introduced into *E. coli* BLR(DE3) harbouring pCondonPlus plasmid (Agilent Technologies) for overexpression of the proteins. hPol η, hPol ι and hPol κ were expressed and purified using the same protocol used for yPol η purification (49), except that protein expression was induced by IPTG for 15–16 h at 18°C, and Q-Sepharose and SP-Sepharose were used as the second columns for hPol η and hPol κ purification, respectively. yRev1 was purified similarly using Ni-Sepharose, SP-Sepharose, and Heparin-Sepharose columns. Protein concentrations were determined based on the band intensity on an SDS-PAGE gel with BSA standard (Pierce). Newly prepared proteins for this study were analysed by SDS-PAGE (Supplementary Figure S1A).

### Extension of labelled primer by DNA polymerase

Extension of ^32^P-labeled primer by DNA polymerase was analysed essentially as described (48). In the standard reactions, a ^32^P-labeled 34-mer primer (TSO590; 0.1 pmol) was annealed with 0.11 pmol of a template DNA and incubated first with yPol δ (0.1 pmol) for 10 min at 37°C in 10 μl of 25 mM Tris-acetate (pH7.5), 50 mM NaCl, 4 mM MgCl_2_, 100 μg/ml BSA, 5 mM DTT, 1 mM ATP and 100 μM each of four dNTPs. Then, 0.1 pmol of a single TLS polymerase (in 0.5 μl), or premixed TLS polymerases (0.1 pmol each) was added to the reaction and incubation continued. A small aliquot was withdrawn from the reactions at indicated times and mixed with 1.5-fold volume of stop buffer (20 mM EDTA, 0.1% bromophenol blue, 0.1% xylene cyanol in formamide). Samples were heated at 95°C for 5 min and separated by electrophoresis through a 10% polyacrylamide gel (25 × 14.5 cm) in TBE buffer containing 7 M urea. ^32^P-labeled DNA products were visualized with a BioRad Molecular Imager Personal FX and quantified with Quantity-One software.

### Quantification of nucleotide misincorporation

A ssDNA template (0.11 pmol) was annealed with an NGS primer (0.1 pmol; Figure 2A) containing a unique barcode by incubating at 94°C for 4 s and gradually cooling to 37°C over 30 min in the same buffer used for the primer extension. When dsDNA template was used (Figures 4–6), annealing was carried out under the same conditions as above except that 1 pmol of the competitor DNA was added to titrate the top strand of the dsDNA template. After the annealing, yPol δ (0.1 pmol in 0.48 μl) was added and incubated for 30 min at 37°C. Then, when indicated, 0.1 pmol of a TLS polymerase (in 0.5 μl), or premixed TLS polymerases (0.1 pmol each) was added to the reaction and incubation continued for another 30 min. The reaction volume including polymerases was 10 μl at this point. When indicated, a dsDNA template was incubated with 2.5 units (0.5 μl) of uracil DNA glycosylase (New England Biolabs) at 37°C for 30 min before heating with the primer and the competitor. For each condition, DNA synthesis reactions were repeated with seven different templates (template A–G). Reaction was stopped by adding 1 μl of 0.5 M EDTA. DNA was extracted by phenol/chloroform/ isoamyl alcohol treatment, precipitated with ethanol, and suspended into 10 μl of H_2_O. Samples were then pooled, concentrated 10-fold by ethanol precipitation, and directly analysed by Ion Torrent Personal Genome Machine (Thermo Fisher Scientific). All output read sequences in FASTAQ format were automatically sorted by barcodes.

Sequence data were processed using Galaxy tools (https://usegalaxy.org/), as described (48). Essentially, low quality reads were removed and base changes were mapped using the Lastz sequence alignment tool (50). Lastz output data were further analysed by Microsoft Excel. To distinguish primer extension products from undesired template extension products, the NGS primers have a mismatched base at the centre of the primer-template hybridization site (10G; Figure 2A). Lastz output were screened for 10G and all reads containing 10G were defined as qualified reads. The numbers of the qualified reads (n) are shown in Supplementary Table S2. The misincorporation frequency (%) was calculated for individual bases of the reference sequence. Therefore, all mutation frequencies were calculated as % of mutations at individual bases in full-length primer extension products. Background frequencies at individual bases were obtained from the same reactions on the unirradiated ssDNA templates and subtracted from the data obtained with irradiated samples. To analyse sequence contexts, the mutation frequencies of individual bases in the 50-nt regions (position #22–71) of template A-G (total 350-nt) were sorted by sequence context and average mutation frequencies with SD were calculated. Cosine similarity between two trinucleotide mutation spectra was calculated from cosine distance of their 96-dimension vectors. Cosine distance was calculated using the dist.cosine function of R (51).

### Statistical analysis

Details of statistical analyses of individual experiments including ‘n’ are described in the figures and figure legends. The numbers of the qualified reads (*n*) for individual NGS samples are shown in Supplementary Table S2. GraphPad Prism version 6.07 was used to compute statistic values.

## RESULTS

### *In vitro* TLS on UV-irradiated template DNA

**Figure 1.**
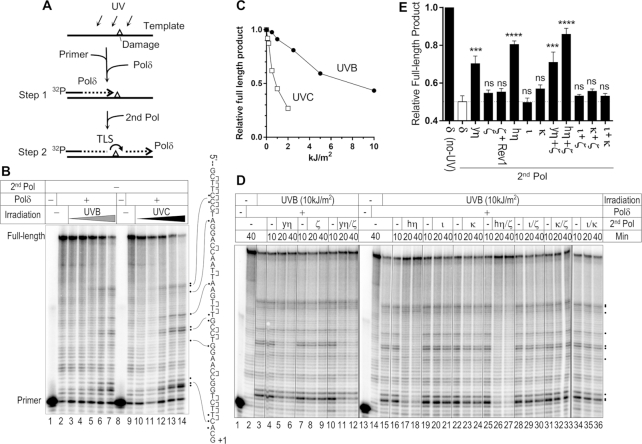
TLS by yPol η, hPol η, yPol ζ, hPol ι, and hPol κ on UV-irradiated template. (**A**) Illustration of experimental design. See main text for explanation. (**B**) Primer extension by yPol δ alone. A ssDNA template (Figure 2A) was irradiated with UVB (302 nm; 0, 0.5, 1, 2.5, 5 and 10 kJ/m^2^ in lanes 2–7) or UVC (254 nm; 0, 0.1, 0.2, 0.5, 1, and 2 kJ/m^2^ in lanes 9–14), and used in primer extension by yPol δ. Products were analysed by electrophoresis through a polyacrylamide gel containing 7M urea (sequencing gel). Potential sites of pyrimidine dimers (indicated by ‘]’) and major sites that blocked the extension (dots) were indicated on the template sequence. (**C**) Quantification of relative amount of full-length DNA products from panel B. (**D**) Primer extension experiments were carried out using yPol δ and the indicated second DNA polymerases (second Pol; ‘yη’ = yPol η, ‘ζ’ = yPol ζ, ‘hη’ = hPol η, ‘ι’ = hPol ι, ‘κ’ = hPol κ). Samples were withdrawn from the reaction at indicated times and analysed by the gel electrophoresis. (**E**) Similar experiments to D with indicated second DNA polymerases were repeated and relative formation of full-length product after 30 min of reaction were calculated (mean + SEM, *n* = 3, ***P < 0.001, ****P < 0.0001, ^ns^*p* ≥ 0.05; One-way ANOVA with Bonferroni's multiple comparison test to the ‘δ’ data that is shown as a white bar).

We characterized the TLS activity of purified polymerases on a UV-irradiated template (Figure 1A). A 99-mer ssDNA molecule was irradiated with UV and hybridized with ^32^P-labeled primer and mixed with yeast Pol δ (yPol δ) to initiate primer extension. Since yPol δ is a high-fidelity replicative polymerase, primer extension should stop at damaged bases in the template (Figure 1A, step 1). As expected, increasing doses of UVB (302 nm) and UVC (254 nm) irradiation stopped primer extension by yPol δ at several template sites one nucleotide before contiguous pyrimidines (dipyrimidines; Figure 1B). Full-length products were reduced ∼50% when the template was irradiated with 10 kJ/m^2^ of UVB (Figure 1C). UVC was ∼10 times more effective than UVB in blocking primer extension, consistent with previous reports that quantified UV-induced DNA damage (reviewed in (1)).

Although UVC effectively creates pyrimidine dimers it is mostly absorbed by atmosphere. UVB is more abundant in solar light that reaches the ground. Therefore, we used a template ssDNA that was irradiated with 10 kJ/m^2^ of UVB to test the TLS activity of yeast Pol η (yPol η), human Pol η (hPol η), yeast Pol ζ, (yPol ζ), human Pol ι (hPol ι) and human Pol κ (hPol κ) (Figure 1D and E). After 10 min of primer-extension by yPol δ, a second polymerase (second Pol) was added and incubation continued to test for TLS activity (Figure 1A, step 2). In the absence of second Pol, irradiated template blocked primer extension by yPol δ at the dipyrimidine sites of the template (Figure 1D, lanes 4, 7, 10, 16, 19, 22, 25, 28, 31 and 34). When yPol η (yη; lane 4–5) or hPol η (hη; lane 17–18) was used as a second Pol, the shorter products were mostly diminished and the full-length product was significantly increased (Figure 1D and E), indicating that both yPol η and hPol η can mediate TLS on the UV-damaged template.

yPol ζ, which has been reported to efficiently extend mismatched 3′-ends, slightly increased the full-length product when mixed with yPol η (Figure 1E, ‘yη+ζ’) and hPol η (‘hη+ζ’). The significance of this stimulatory activity in our system was not evident. yPol ζ alone had only minor TLS activity and yeast Rev1 (yRev1) did not stimulate TLS when mixed with yPol ζ. hPol ι did not have significant TLS activity under our conditions. hPol κ had moderate but much lower TLS activity than hPol η or yPol η. Quantitative representation of repeated experiments (Figure 1E) showed that significant TLS activity was consistently observed when the hPol η or yPol η was used as the second polymerase. Standalone DNA polymerase activity was confirmed for all polymerases used in the primer extension experiments (Supplementary Figure S1B).

### TLS misincorporates nucleotides on UV-irradiated ssDNA template

**Figure 2.**
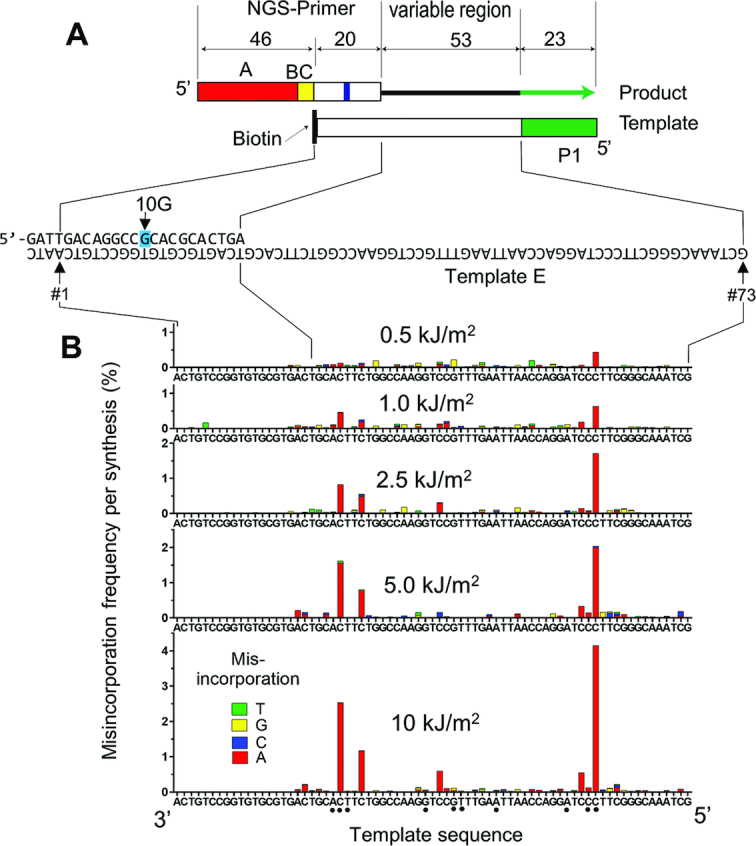
TLS-associated nucleotide misincorporation on UVB-irradiated ssDNA template. (**A**) Illustration of a synthetic DNA substrate used to quantify TLS-mediated nucleotide misincorporation. The NGS-primer contained an A-adaptor (red box) and a barcode (yellow box, BC) while the template contained a P1-adaptor (green box) for Ion Torrent NGS. Only the primer extension products that contain both adaptors are sequenced, reading a 73-nt region (position #1–73), in which 20nt (#1–20) belong to the primer and 53nt (#21–73; variable region) are produced by primer extension. The NGS primer had a one-base mismatch at position #10 (10G) to distinguish the primer-extension products from template-extension products. (**B**) The ssDNA template was irradiated with the indicated dose of UVB, then hybridized with NGS primer containing a unique barcode and subjected to primer extension reaction first by yPol δ and then by premixed yPol η + yPol ζ. Full-length products were directly analysed by NGS, and single nucleotide misincorporation frequencies per full-length product are indicated by bars (green:T; yellow: G; blue: C; and red: A).

We quantified nucleotide misincorporation on UV-irradiated ssDNA template using the NGS-based method described in our previous work (Figure 2) (48). The same template used in Figure 1 (template-E) was irradiated with UVB and subjected to primer extension from a primer that contained an adaptor sequence for NGS (Figure 2A, ‘NGS primer’). The template contained the P1 adaptor sequence, allowing the full-length product to be sequenced by NGS. The 3′-OH of the template was modified with a biotin to suppress extension of the template strand. Since this 3′ biotin modification might be removed by the proofreading exonuclease activity of yPol δ, one mismatch (10G) was introduced in the NGS primer to distinguish primer-extension products from template-extension products. A small fraction (generally 1–5%) of the reads did not have 10G and were excluded from further analyses. We carried out individual TLS reactions using primers with unique barcodes and the resulting DNA products were pooled after reactions were stopped. The pooled DNA samples were subjected to parallel sequencing without preceding amplification to quantitate nucleotide misincorporation frequency.

When premixed yPol η + yPol ζ were used as TLS polymerases, misincorporation occurred in the primer-extension products in a UV dose-dependent manner (Figure 2B). The majority of the UV-induced base changes were misincorporations of dAMP opposite of a C residue in dipyrimidine sites, creating C>T transitions. Among the dipyrimidine sites, TpC template sites showed the highest misincorporation frequencies, varying from 1% to 4% of the full-length primer extension products, when the template was irradiated with 10 kJ/m^2^ of UVB. The TLS products had almost no detectable misincorporation at TpT template sites, confirming the error-free TLS activity of Pol η over TT dimers.

### Similarity of the TLS-associated base substitution spectrum to a skin cancer mutation signature

**Figure 3.**
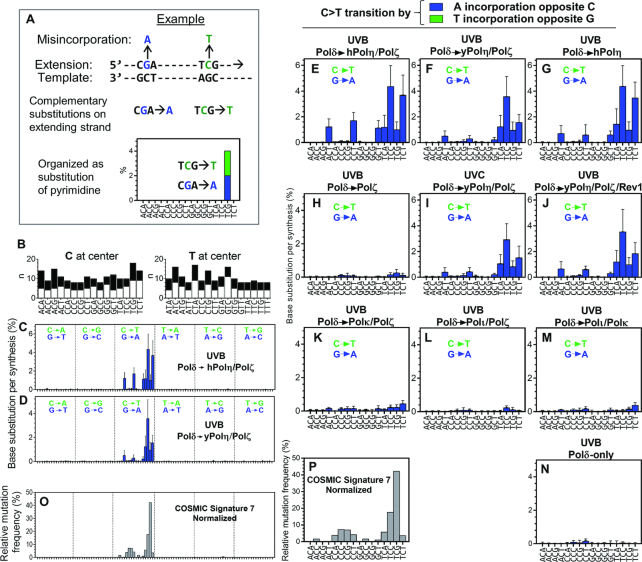
Base substitution spectra of TLS-products. (**A**) An example to explain the analysis. In this example, two nucleotide misincorporations (blue and green) on the extending strand are complementary to each other, thus they are not distinguished in genome sequencing. Since our *in vitro* approach separately quantify frequencies of these substitutions, their frequencies are shown at the same position of graph by blue bar (purine mutation) and green bar (pyrimidine mutation) of the extending strand. (**B**) Numbers of appearances (n) of indicated trinucleotide sequences (white bars) and their complementary sequences (black bars) in the extending strand of the seven templates (Supplementary Figure S2A; template A-G, total 350-nt). (**C** and **D**) Template A-G were irradiated with UVB (10 kJ/m^2^) and used for TLS reactions by yPol δ and indicated TLS polymerases. Products were analysed by NGS and the misincorporation frequencies are presented in the 96-trinucleotide format used for the cancer mutation signatures (5), with two exceptions. First, the vertical axis is frequency per full-length product (mean +SD, n is shown in (B)). Second, complementary substitutions observed in the extending strand are expressed in green (change of pyrimidines) and blue (change of purines) bars, as explained in (A), although green bars are mostly invisible in this Figure. (**E**–**N**) Spectra of C>T transition frequencies in the primer extension products produced in the absence or the presence of indicated second DNA polymerase. The templates were irradiated with UVB (10 kJ/m^2^) or UVC (2 kJ/m^2^) as indicated. Entire 96-dimensional spectra are shown in Supplementary Figure S3. (O and P) Normalized Cosmic Signature 7 (**O**) and its C>T portion (**P**) (5).

To further investigate TLS-associated mutagenesis, we irradiated seven ssDNA templates with UVB (10 kJ/m^2^) or UVC (2 kJ/m^2^) and measured TLS-associated nucleotide misincorporation in the presence and absence of TLS polymerases. These templates (template A-G; Supplementary Figure S2A) had randomly chosen sequences in the 53-nt variable region except that homomultimers (e.g. ApA or CpCpC) exist only in template E, F and G. Nucleotide misincorporation frequency (% in full-length products) was calculated at each nucleotide of templates (Supplementary Figure S2B shows some examples). Frequency data within the variable region, excluding the nucleotide adjacent to the primer and two last nucleotides of the region (total 50-nt region × 7 templates = 350-nt), were used to generate a mutational spectrum. The misincorporations were sorted by the trinucleotide context (triplet) format that is used to express cancer mutational signatures (Figure 3C–N and Supplementary Figure S3). In the previous large-scale genomic sequence analyses, complementary mutations (for example CGA>A and TCG>T, see Figure 3A) are indistinguishable. Therefore, the data presented in those reports lists both CGA and TCG triplets together, and mutations are expressed as change of pyrimidines. We have organized our data similarly for comparative purpose. Frequencies of two complementary substitutions in the extended strand (see Figure 3A and legend as an example) were added together (in different colors) in a column and the triplet below each column has pyrimidine at its centre.

There is a significant similarity between the mutational spectra of the TLS products made in the presence of hPol η + yPol ζ (Figure 3C) and of yPol η + yPol ζ (Figure 3D; cosine similarity = 0.92). More importantly, these spectra showed certain similarity (cosine similarity = 0.48 and 0.58 for Figure 3C and D, respectively) to the predominant mutational signature in melanomas after normalization of trinucleotide frequency of the human genome (Cosmic Signature 7 normalized; Figure 3O). The vast majority of the single nucleotide substitutions produced by the hPol η + yPol ζ combination (Figure 3C) and by the yPol η + yPol ζ combination (Figure 3D) are C>T (G>A) transitions (96% and 94%, respectively). This is consistent with Signature 7, in which 96% of mutations were C>T transition (5). Our *in vitro* analysis separately analysed C>T (green bars) and G>A (blue bars), but no C>T was detected on the extending strand, indicating that practically all substitutions were created by misincorporation of dAMP opposite C template residues that likely formed pyrimidine dimers. Figures 3E, F and P, which magnify C>T transition frequencies of Figure 3C, D and O, respectively, clarify that in both *in vitro* spectra and Signature 7, C>T transition frequencies were above the background level in TpCpN and NpCpT contexts. However, the cancer signature shows remarkably high rate in TpCpG context, which is much less pronounced in Figure 3E and F. This motif is the most frequent site of C deamination after UV exposure (1,52,53) and we will return to this point in the next section. In addition, Signature 7 has moderate levels of C>T transitions at CpCpG and CpCpC contexts, which were almost undetectable in *in vitro* TLS products.

The spectrum was not significantly changed when yPol ζ was not present in the reaction (Figure 3G), but the misincorporation was greatly reduced in the absence of Pol η (Figure 3H), indicating that the most of the misincorporations were introduced by Pol η. The spectrum created by UVC-irradiation (Figure 3I) was not significantly different from that created by UVB (Figure 3F). We found only minor increases in single nucleotide insertions and deletions produced in the presence of hPol η + yPol ζ on UVB-irradiated templates (Supplementary Figure S2C). We also examined the misincorporation spectra in the presence of other y-family polymerases (Figure 3J-M and Supplementary Figure S3). The mutational spectrum produced by yRev1 + yPol η + yPol ζ showed no significant difference from that produced by yPol η + yPol ζ (compare Figure 3F and J). As expected from their lower TLS efficiency (Figure 1), hPol ι and hPol κ showed only minor increases in mutation frequency above background level (Figure 3K–M and Supplementary Figure S3).

### Cytosine deamination-dependent mutations is produced by repeated UV-irradiation and intervening incubation

**Figure 4.**
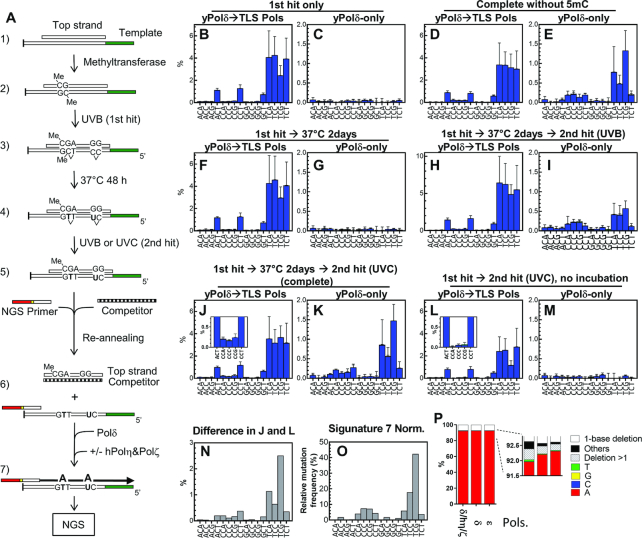
Base substitution spectra created by the ‘two-hit’ deamination procedure. (**A**) A schematic drawing of *in vitro* mutational process to deaminate C and 5mC. First, the template was annealed to its complementary strand (1) and treated with methyltransferase (2). Then it was irradiated with 10 kJ/m^2^ of UVB (3, ‘1st hit’) and incubated at 37°C for 48 h to allow deamination to occur (4). The DNA was irradiated again (‘2nd hit’) by 10 kJ/m^2^ of UVB or 1 kJ/m^2^ of UVC to reverse photodimers (5). The substrate was heat-denatured and reannealed with the NGS primer and excess competitor DNA that titrated the top strand but could not hybridize with the primer (6). Then the primer was extended by yPol δ-only or by yPol δ followed by premixed hPol η + yPol ζ (7). The full-length products were analysed with NGS. (**B**–**M**) The dsDNA substrates were processed as indicated and subjected to the primer extension only by yPol δ (C, E, G, I, K, and M) or first by yPol δ and followed by premixed hPol η + yPol ζ (B, D, F, H, J, and L). Products were analysed by NGS and resulted misincorporation frequencies were sorted into the trinucleotide format (Supplementary Figure S4), and C>T transition frequencies are shown. (**N**) Contribution of the 2-day incubation was obtained by subtracting the frequency data of L from that of J. (**O**) The Signature 7 for visual comparison. (**P**) Primer extension was carried out on a template ssDNA containing a uracil residue (TSO845; see Table S1) by indicated polymerases, and the ratio of nucleotides incorporated opposite uracil was calculated as described (48).

Next, we reproduced another potential mutagenic process, deamination of the cytosines in pyrimidine dimers, as illustrated in Figure 4A. Because this mutagenic process may be influenced by 5mC, template ssDNA was first annealed with an oligo (‘Top strand’) that was complementary to the entire variable region, and the dsDNA template was treated with M.*Sss*I methyltransferase to modify C to 5mC specifically at CpG sites. After confirming C-methylation (Supplementary Figure S6), the DNA was irradiated with 10 kJ/m^2^ of UVB (‘1st hit’) and incubated at 37°C for 48 h in the dark to facilitate deamination. Then we induced monomerization of pyrimidine photodimers by a second exposure of UV-radiation (photoreversion), a process observed in photochemistry (2). Although it is known that CPD can be efficiently monomerized by photolyase activity (54), this enzyme has not been found in humans. Since CPD formation is reversible by UVC (55,56), we irradiated the DNA again with 1 kJ/m^2^ of UVC or 10 kJ/m^2^ of UVB (‘2nd hit’) to simulate mutational process in human cells. After completing the entire process (two-hit deamination) or each step of the process as indicated in the Figure 4B–M, DNA was recovered, heat-denatured, and annealed with the NGS primer in the presence of excess competitor DNA (Figure 4A) to titrate out the top strand. Then the primer was extended only by yPol δ, or first by yPol δ and then by a mixture of hPol η + yPol ζ, and analysed by NGS.

When the templates were exposed to the two-hit deamination procedure (the 1st hit with UVB → incubation at 37°C → the second hit with UVC), significant C>T transitions, especially in the TpCpG context, were observed in the products produced by yPol δ in the absence of TLS polymerases (Figure 4K and Supplementary Figure S4E). This TLS polymerase-independent misincorporation was not observed when the second hit or the intervening incubation was omitted (Figure 4G and M, respectively), indicating that both two hits of UV and an intervening incubation were required for this mutagenic process. Interestingly, the base substitution spectrum (Figure 4K and Supplementary Figure S4E ‘yPol δ-only’) was significantly similar to the Signature 7 (cosine similarity = 0.92 in 96 dimensions).

Mutational spectra created in the presence of premixed hPol η + yPol ζ (‘yPol δ→TLS Pols’) were also influenced by the two-hit treatment. Spectra produced by the complete two-hit treatment with and without intervening incubation (Figure 4J and L, respectively) showed a significant difference (*P* < 0.0001), indicating that the intervening incubation increased the C>T transition frequency in the presence of TLS polymerases. The impact of the incubation, which was calculated by subtracting the data presented in Figure 4L from Figure 4J (Figure 4N), is very similar to the yPol δ-only spectrum created on the templates after the complete two-hit treatment (Figure 4K; cosine similarity = 0.98). These results indicate that the incubation after the 1st hit produces modified bases that become available after the second hit for the yPol δ-dependent primer extension, which creates the C>T transitions. Such modifications are very likely the deamination of C residues.

Another notable observation was that C>T transitions in CpCpV (V is A, C or G) context were observed at similar frequencies in the presence or absence of the TLS polymerases (compare insert in Figure 4J with Figure 4K), suggesting that these transitions were mainly created by yPol δ even in the presence of TLS polymerases. This suggests that CC dimers did not cause the single nucleotide substitutions unless deaminated and monomerized.

### Only TLS produces dinucleotide substitution from CC to TT

**Figure 5.**
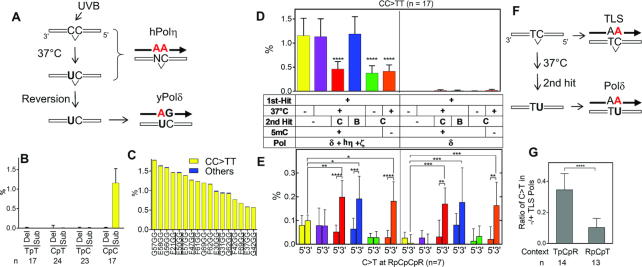
Single and multinucleotide substitutions at CC photodimers. (**A**) A schematic of proposed mutational processes at CC dimers. hPol η inserts two dAMPs at the template dimer regardless the deamination status. After the photoreversion, yPol δ can insert dAMP opposite U, which was created only at 3′ side of the CC dimer. (**B**) Frequencies of multinucleotide deletions (Del) and multinucleotide substitutions (Sub) that were produced at indicated dipyrimidine template sites on the ‘1st hit only’ template in the reaction containing yPol δ, hPol η, and yPol ζ (shown in Figure 4B). Mean +SD are shown (*n* of each dipyrimidine sites are indicated under the graph). (**C**) Frequencies of dinucleotide substitutions at individual CpC template sites produced in the same reaction as (**B**). Yellow bars are CC>TT substitutions, and blue bars (although barely recognizable) are all other multinucleotide substitutions. (**D**) Frequencies of the CC>TT substitutions in the same reactions as in Figure 4B-M (mean + SD, *n* = 17, ****P < 0.0001; One-way ANOVA with Dunett's multiple comparison test to the left-most column in each section). Experimental conditions are indicated under the graph, in which the 2nd hit was made by either UVB (‘B’) or UVC (‘C’). (**E**) Frequencies of the C>T transition at 3′C or 5′C of CpC sites in the corresponding reactions in (**D**). Only CpC sites that had no adjacent pyrimidine (RpCpCpR context where R = purine, underlined in (**C**)) were analysed (mean +SD (*n* = 7), *P < 0.05, **P < 0.01, ***P < 0.001, ****P < 0.0001; two-way ANOVA with Dunett's multiple comparison test). (**F**) Illustration of CT dimer to produce C>T transition by either TLS or deamination. (**G**) The ratio of C>T transition frequencies in the absence/presence of premixed hPol η + yPol ζ (TLS Pols) was calculated in two sequence contexts (TpCpR and RpCpT, where R = purine) from data in Figure 4K and J (mean +SD (n is indicated under the graph), ****P < 0.0001; two-tailed unpaired *t*-test).

The genomes of human melanoma cells have a large number of CC>TT dinucleotide substitutions (5,6,23). Consistently, TLS on irradiated templates produced high frequencies of multinucleotide substitutions at CpC template sites (Figure 5B), and the vast majority (97–100%) of these multinucleotide substitutions were misincorporations of two AMPs, equivalent to CC>TT dinucleotide substitutions (Figure 5C, yellow bars). We next analyzed the CC>TT frequecnies following the deamination and photoreversion (Figure 5D). The 2nd hit by UVC (red, green, and orange bars), but not UVB (blue bar), reduced the CC>TT frequency, confirming that UVC mediated photoreversion of the CC dimer. There were very few CC>TT substitutions in the yPol δ-only reactions under any condition tested (Figure 5D, right half of the graph), indicating that this dinucleotide substitution was mainly created by TLS activity incorporating two dAMPs at the CC dimers, as illustrated in Figure 5A.

We next analysed the single nucleotide C>T transition at CpC sites, which were specifically produced at CC dimers (Figure 5E). To eliminate the influence of overlapping pyrimidine dimers, we analysed only CpC sites that had no adjacent pyrimidine (n = 7; sites are underlined in Figure 5C). The frequency of dAMP single nucleotide incorporation (equivalent to C>T transition) was elevated at the 3′C of the CpC template sites treated by the complete two-hit deamination (Figure 5E, red bars). The lack of intervening incubation (green bar) or second hit (purple bar) eliminated elevated dAMP misincorporation, confirming that these misincorporations were the result of C deamination in the template. A second hit by UVB (blue bars), which did not reduce the CC>TT frequency (Figure 5D), showed elevated misincorporation at the 3′C (Figure 5E), indicating that the UVB resolved deaminated CC dimers but also produced new CC dimers, making the total number of CC dimers constant. In addition, elevated 3′C>T transition was also observed in the absence of TLS polymerases (right half of Figure 5E), confirming that the complete two-hit treatment induced deamination of the 3′C in CC dimers, monomerized the CU dimer to produce free uracil residues that were used as a template for yPol δ-dependent DNA synthesis (Figure 5A).

In contrast, the 5′C in the template CpC sites showed no significant increase in the dAMP misincorporation after the complete two-hit deamination procedure, although photoreversion of CC dimer should produce the same number of monomeric 5′C and 3′C residues. To determine if this 3′-bias in C deamination occurs in TC and CT dimers, C>T transition frequencies at RpCpT and TpCpR contexts were extracted from the data in Figure 4J and K, where templates were treated by the complete two-hit deamination procedure. Because both TLS and the deamination results in C>T transition at TC and CT dimers (Figure 5F), we took the ratio of C>T transition frequencies of yPol δ-only products divided by that of the TLS products (Figure 5G). The ratio was significantly higher in the TC dimer than in the CT dimer, indicating that a higher fraction of C in TC dimers was deaminated than those in CT dimers. Potential mechanisms leading to the 3′-bias are presented in the Discussion.

### Deamination of C was amplified by multiple repeats of UVB irradiation and incubation.

**Figure 6.**
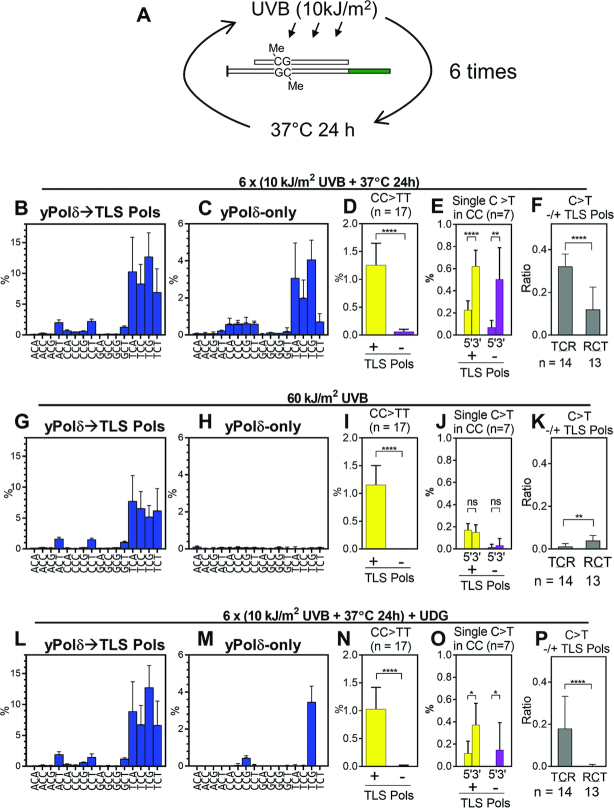
Base substitution spectra produced by six repeats of UVB exposure and 37°C incubation. (**A**) A schematic of the repeated UVB-irradiation and incubation experiment. Template dsDNA (template A–G) containing 5mC was irradiated with 10 kJ/m^2^ of UVB and incubated at 37°C for 24 h in the dark, and this process was repeated total of six cycles. (**B**–**F**) After the sixth irradiation, DNA was recovered and used for primer extension by yPol δ followed by premixed hPol η + yPol ζ (TLS Pols; B), or only by yPol δ (C). The C>T substitution spectra (B and C), CC>TT substitution in the presence or the absence of TLS polymerases (D), and C>T substitution at 5′C and 3′C of RpCpCR context in the presence or the absence of TLS polymerases (E), the ratio of C>T substitution frequencies in the absence/presence of TLS polymerases at TpCpR and RpCpT sites (F) were calculated as described above. (**G**–**K**) Same as panel B–F except that templates were irradiated with 60 kJ/m^2^ in a single step without intervening incubation. (**L**–**P**) Same as panel B–F except that templates were treated with UDG after sixth irradiation. All data are presented as mean +SD (*P < 0.05, **P < 0.01, ***P < 0.001, ****P < 0.0001, ns = not significant; two-tailed unpaired *t*-test).

When UVB (10 kJ/m^2^) rather than UVC (1 kJ/m^2^) was used for the 2nd hit (Figure 4I), yPol δ-dependent misincorporation was induced but to a lesser extent. This result is consistent with previous observations that pyrimidine photodimers poorly absorb UVB light (12,55,57). Although UVC effectively induces photoreversion, practically no UVC component in solar light reaches the ground. To analyze C-deamination-dependent mutational spectrum using only UVB radiation, we repeated the irradiation-incubation cycle six times (Figure 6A). The UVB source used in this study was not monochromatic but had much <1% of its total energy in UVC range and most of the residual UVC light was blocked by a filter. The repeated treatment clearly increased the frequency of misincorporation in the primer extension products by yPol δ (Figure 6C and Supplementary Figure S7). In addition, the yPol δ-mediated base substitution spectrum was very similar to that produced by a single second hit with UVC (Figure 4K) and to Signature 7 (cosine similarity = 0.99 and 0.89, respectively). Although we could not draw a quantitative conclusion about UVB-mediated photoreversion efficiency, these results suggest that multiple rounds of exposure of UVB or very small dose of UVC could induce a mutation spectrum that is similar to Signature 7.

The same total dose of UVB (60 kJ/m^2^) without intervening incubations did not induce any significant yPol δ-mediated misincorporation (Figure 6H), confirming that time-dependent deamination of C was involved in the mutational process. Although the total incubation time was 5 days, the vast majority of the CC>TT substitutions were TLS-dependent and very few were produced in yPol δ-only reactions (Figure 6D), indicating that the prolonged incubation did not facilitate the double deamination of CC dimers. The 3′-bias in deamination of CC dimers was also observed (Figure 6E). Cytosines in TC dimers were more readily deaminated than in CT dimers (Figure 6F). These results indicate that repeated exposures to UVB with intervening incubation at 37°C can reproduce the deamination-mediated mutational process. To confirm that the templates contained U and T residues that were created by the deamination of C and 5mC, respectively, we treated the template with uracil-DNA glycosylase (UDG) after the sixth UVB treatment (Figure 6L–P). The UDG-treatment almost completely abolished C>T transitions in the yPol δ-only products except for NpCpG template sites that were the target of the CG-methyltransferase (Figure 6M), confirming our conclusion that the yPol δ-dependent nucleotide misincorporation was caused by cytosine deamination.

### Influence of 5-methyl cytosine

To evaluate the influence of 5mC, we omitted the CpG methyltransferase from the two-hit deamination procedure and examined single nucleotide substitution spectra (Figure 4D and E), dinucleotide CC>TT substitution (Figure 5D, orange bars), and C>T single nucleotide transition by CC photoreversion (Figure 5E, orange bars). Absence of 5mC had no significant effect on any substitution types tested. In addition, significant difference was not observed in the deamination-mediated C>T transition frequencies when individual TpCpG and CpCpG sites were compared in the presence and absence of the methyltransferase treatment (Supplementary Figure S5). We confirmed the presence of 5mC in the template DNA by resistance to UDG treatment (Figure 6M) and restriction digestion (Supplementary Figure S6).

## DISCUSSION

**Figure 7.**
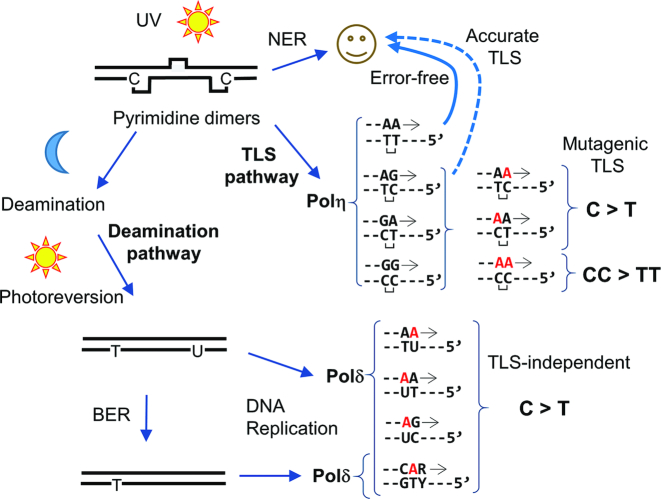
Model of TLS and deamination-dependent mutagenesis pathways. See main text for details.

This paper presents biochemical evidence that supports two proposed *in vivo* pathways of UV-induced mutagenesis that are implicated in human melanoma (Figure 7). One pathway involves TLS over template pyrimidine dimers (TLS pathway) and the other involves the deamination of C in pyrimidine dimers followed by photoreversion (deamination pathway). Both pathways mainly create C>T transitions at template dipyrimidines and have similar single nucleotide substitution spectra, but these mutational processes are fundamentally different. Analysis of mutations derived from our reconstituted TLS pathway (Figures 2–3, and other Figures that use TLS polymerases) show that DNA synthesis on UV-irradiated templates by Pol η create (i) no error at TT photodimers, (ii) C>T transitions at TC and CT photodimers and (iii) CC>TT dinucleotide substitution at CC photodimers. These results indicate that Pol η can mediate error-free and mutagenic TLS by the same mechanism, in which two dAMPs are incorporated opposite of the photodimers (Figure 7, ‘TLS pathway’). This phenomenon has been known previously as ‘A-rule’ (58), in which damaged template bases are most frequently paired with A. We also reconstituted deamination-mediated mutagenesis *in vitro*, by irradiating dsDNA with UVB, then incubating at 37°C for 24–48 h to allow C-deamination, and then irradiating again with UVC or UVB (Figure 7, ‘Deamination pathway’). Our results (Figure 4) indicate that the first UV-exposure (first hit) creates pyrimidine dimers, and incubation at 37°C leads to deamination of C to U and 5mC to T in pyrimidine dimers. The second UV-exposure (second hit) then monomerizes the dimers. Once monomerized, the U residue poorly blocked DNA synthesis by yPol δ, which incorporated dAMP at the site (Figure 4P). This process creates C>T transitions.

How do these pathways contribute to mutational processes that lead to skin cancer? Although our *in vitro* results do not constitute biological evidence for causation of cancers, they suggest that both pathways can contribute to mutational processes that lead to UV-induced skin cancers. The high similarity of the mutational spectrum of the deamination pathway examined here to the Signature 7 (cosine similarity = 0.89–92), provides strong circumstantial evidence that C-deamination is the predominant pathway in skin cancer development. CC>TT dinucleotide substitution, which is another ‘signature mutation’ of skin cancers (5,6,23), was generated only by the TLS pathway in our experiments (Figures 5D and 6D). This result suggests that TLS is also involved in skin cancer development. The spectra generated by the TLS pathway were generally less similar to Signature 7 than of deamination pathway, but high similarity (cosine similarity = 0.83–0.85) was obtained when the templates were irradiated by UVB six times and incubated at 37°C for 24 h between UV-exposures (Figure 6B and L). Therefore, it is likely that the two pathways additively contribute to the mutations observed in melanomas. Obviously, there must be many other factors that influence the UV-induced mutational process *in vivo* that were not included in this study. For example, the PCNA sliding clamp and its mono- and poly-ubiquitination, SUMOylation and acetylation derivatives (59,60), the DNA repair systems (61,62), sequence-specific DNA binding proteins, and chromatin context (63–65) would likely influence both pathways and affect mutational spectra and overall mutation frequencies in cancers.

Numerous reports and data presented here demonstrate that Pol η can mediate error-free bypass of TT dimers. However, while our results show that hPol η mediates mutagenic TLS at C-containing pyrimidine dimers, previous reports indicate that hPol η reduces UV-induced mutagenesis at TpC and CpC sites, most likely by ‘accurate TLS’ (24,25), as indicated with a dashed arrow in Figure 7. We believe that accurate and mutagenic TLS are not mutually exclusive. It is possible, even likely, that fidelity of hPol η-mediated TLS across C-containing dimers is not perfect. In addition, since our experiments used template DNA that was randomly damaged by UV exposure, mutation frequency per pyrimidine dimer was not measured. We propose that although many C-containing pyrimidine dimers might be bypassed by accurate TLS, at least a fraction of TLS products contain misincorporations and create mutations. Previous studies have indicated that Pol η can distinguish C and U in CPD without reversion of the dimer structure (66,67), suggesting that the mutagenic TLS may be due to Pol η-mediated TLS across deaminated CPD. However, Figure 4B and F show that the deamination without the second hit did not produce significant change in C>T spectrum. This suggests that Pol η inserted AMP opposite C in CPD in the same frequency in our system regardless of deamination status.

Available evidence indicates that hPol η, when present, mediates most of the TLS events that occur in UV-irradiated cells. Therefore, it is reasonable to hypothesize that the mutagenic TLS by hPol η, shown here, is the primary process that creates TLS-associated mutations in normal cells after UV-irradiation. Our results do not exclude the possibility that human cells have other error-prone TLS mechanisms. However, important candidates of such alternative TLS polymerases, hPol ι and hPol κ (16,31,36–42) had much lower TLS activity than hPol η in our system. Therefore, we could not obtain a sufficient number of full-length TLS products that could be analysed by NGS for reliable evaluation of their mutational spectra. This might be because our experiments included yPol δ that had a proofreading exonuclease activity. Although hPol ι and hPol κ might incorporate nucleotides opposite of the lesions, the unpaired 3′-end might be removed by yPol δ. Therefore, our interpretation of the results (Figure 1D and E) is that yPol η and hPol η, but not hPol ι or hPol κ, extended the unpaired 3′-end over the pyrimidine dimer to form a base-paired region that is long enough to overcome the proof reading activity of yPol δ. It is also possible that other factor(s) may be required for these enzymes to mediate efficient TLS *in vivo*. Candidate factors include PCNA and its derivatives, and species-specific interactions of proteins in the reaction. To simulate the mutagenic process, our experiments used irradiated DNA, not defined DNA lesions, and our TLS efficiency did not reach 100% (Figure 1E). Therefore, it is also possible that specific types of lesion might not be bypassed by hPol η + yPol ζ and escape our mutational analysis. Such lesions might be bypassed in the presence of the factors mentioned above or by certain combinations of TLS polymerases that were not tested in this study. Alternatively, some lesions might not be bypassed efficiently and cause cell death if not repaired *in vivo*.

Our experiments detected very little CC>TT dinucleotide substitution in the deamination pathway, even after 5 days of incubation and repeated irradiation (Figure 6D, ‘no TLS Pols’). The majority of the base changes we observed at CC dimers in the deamination pathway were single nucleotide C>T transitions at the 3′C. In addition, C-deamination based on mutation frequencies occurred more frequently in TC dimers than in CT dimers (Figures 5G, 6F and 6P). These data are consistent with genetic observations showing that the 3′C of dipyrimidine site is mutated more often than the 5′C (3,23–25,68). Based on these observations, we speculate that 3′C in pyrimidine dimers is more efficiently deaminated. However, photochemical studies showed that the deamination of the 3′C and the 5′C within dimers occur at similar rates (10–12). These studies measured deamination in dinucleotides, not DNA, without UV-induced photoreversion. Since we measured C deamination by dAMP incorporation by yPol δ after photoreversion, the 3′-bias might be the result of photoreversion or dNMP incorporation. For example, the low CC>TT substitution frequency in the deamination pathway might be due to poor photoreversion efficiency of UU dimer. Alternatively, the 3′-bias might be caused by different nucleotide incorporation frequencies of yPol δ at 3′U and 5′U. It may be interesting to compare mutational spectra using different reversion mechanisms, like UV-induced reversion and photolyase-mediated reversion.

Figure 4 shows that yPol δ has a prominent ability to make C>T transition at TpCpG sequence on deaminated templates. This is not consistent with earlier report showing that C>T at the same context requires Pol η in mice (69). However, this and many other UV-induced mutagenesis experiments in mammalian models have irradiated animals only a single time. Our model (Figure 7) suggests that there was little chance to monomerize photodimers in these experiments. Therefore, the contribution of TLS-associated mutational process might be overestimated in these studies.

The deamination pathway seems to require reversion of pyrimidine dimers. Since humans do not have a functional photolyase, UV-induced photoreversion seems to be crucial for this pathway *in vivo*. However, photochemical studies have shown that photoreversion requires UVC, and that pyrimidine dimers do not absorb UVB light (12,55–57). If the UVC of the solar light does not reach the ground, then how can solar light induce photoreversion? This dilemma could be solved in either of following hypotheses: (i) Solar light has little UVC, but the dose is still sufficient to induce photoreversion, or (ii) pyrimidine dimers poorly absorb UVB, but photoreversion is efficient enough to use this energy. The first hypothesis seems less likely except at extreme geographic locations (e.g. high altitude or thin ozone layer). The second hypothesis is supported by this study (Figure 6). This hypothesis is also supported by a study reporting that C-containing CPDs absorb UVB (70). However, it has not been tested whether sunlight can induce the same reaction. Alternatively, undamaged bases at the close proximity of a pyrimidine dimer, or another molecule in the skin cell might absorbs UVB and function as electron donors to mediate photoreversion (57,70).

## DATA AVAILABILITY

NGS raw data for this work have been deposited with figshare (https://figshare.com) under ‘*Biochemical Reconstitution of UV-induced Mutational Processes*’.

## Supplementary Material

gkz335_Supplemental_Files
