# Supplementary material for: Biochemical reconstitution of UV-induced mutational processes
Source: Nucleic Acids Res. 2019 May 4;47(13):6769–82. doi: 10.1093/nar/gkz335 (PMC6648339; doi:10.1093/nar/gkz335)
Supplement: gkz335_Supplemental_Files [file gkz335_supplemental_files.zip › SuppFigs.pdf]

Supplemental Figures for

Biochemical Reconstitution of UV-induced Mutational Processes

by

Tomohiko Sugiyama and Yizhang Chen

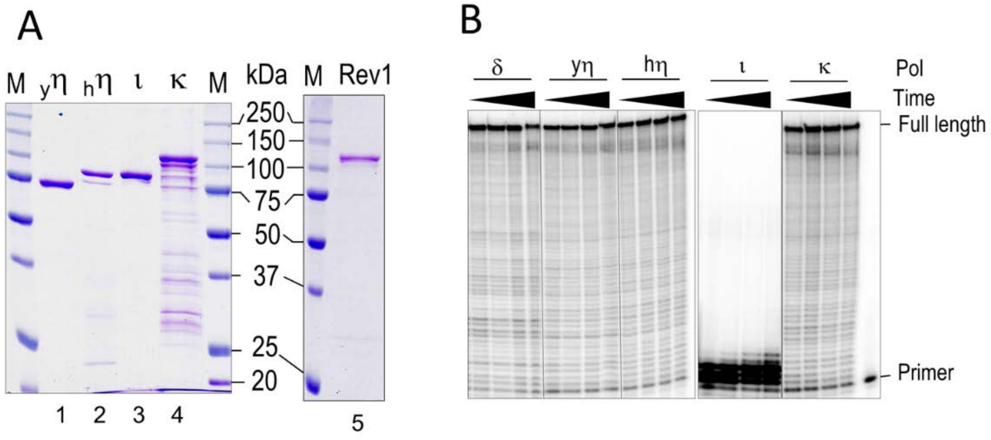

Supplementary Figure S1. Purified DNA polymerases for this study. (**A**) Proteins were separated by SDS-PAGE (10%) with molecular mass marker (M) and stained by coomassie brilliant blue R250. Lane 1- 5 contain, 1 µg of yPol η ($_y$η), 1 µg of hPol η (hη), 1 µg of hPol ι (ι), and 1 µg hPol κ (κ), and 0.51 µg of yRev1. (**B**) Primer extension by indicated polymerase was carried out on a unirradiated template (template A). Reactions were stopped at 5, 10, 20, and 40 min (from left to right) after a polymerase was added, and products were analyzed by denaturing polyacrylamide gel electrophoresis. hPol ι extended the primer for only a few nucleotides but majority of the primers were extended at least 1-nt, consistent with previous reports (1-4).

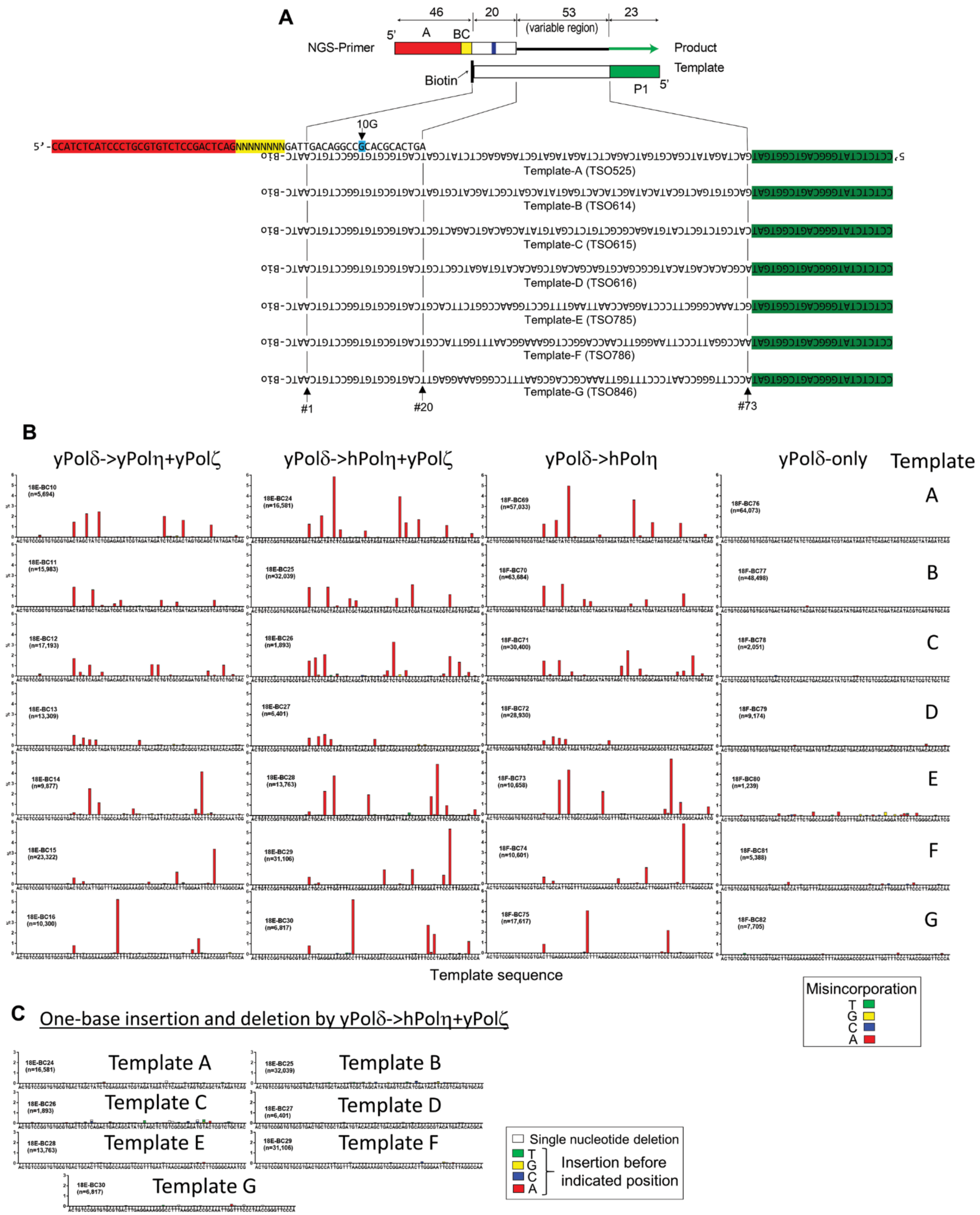

Supplementary Figure S2. Template ssDNA (Template A-G, shown in (**A**)) were irradiated with UVB (10 kJ/m^2) and subjected to the primer extension first by yPol δ and then by indicated TLS polymerases. Products were analyzed by NGS as described in Method, and frequencies of UV-induced single nucleotide misincorporations were in shown in (**B**), and single nucleotide insertion and deletion were shown in (**C**).

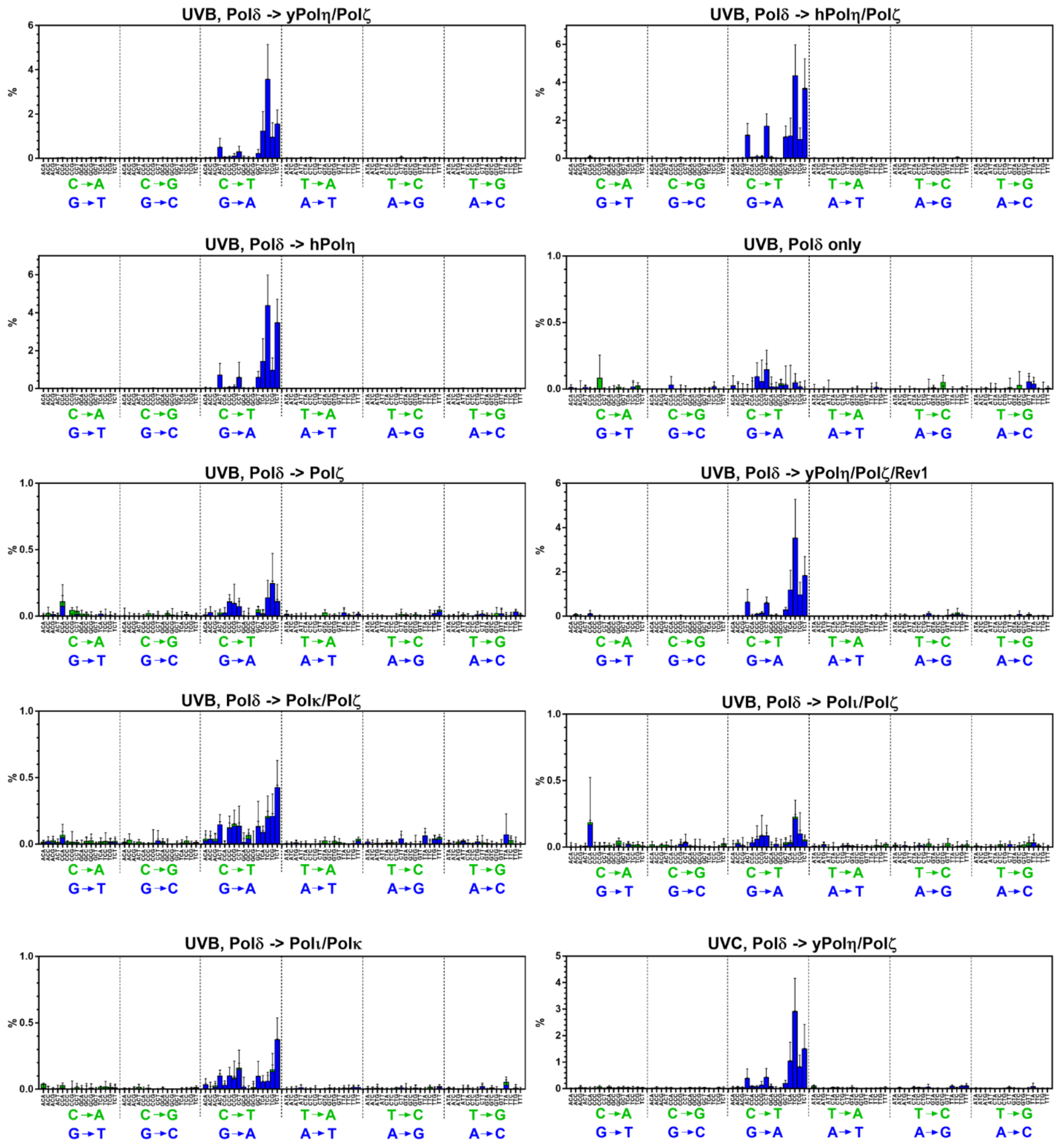

Supplementary Figure S3. Template ssDNA (Template A-G, shown in **Supplementary Figure S2A**) were irradiated with UVB (10 kJ/m^2) or UVC (2 kJ/m^2), and subjected to the primer extension first by yPol δ and then by indicated TLS polymerases. Products were analyzed by NGS and UV-induced nucleotide substitutions spectra were obtained from frequencies of single nucleotide misincorporations.

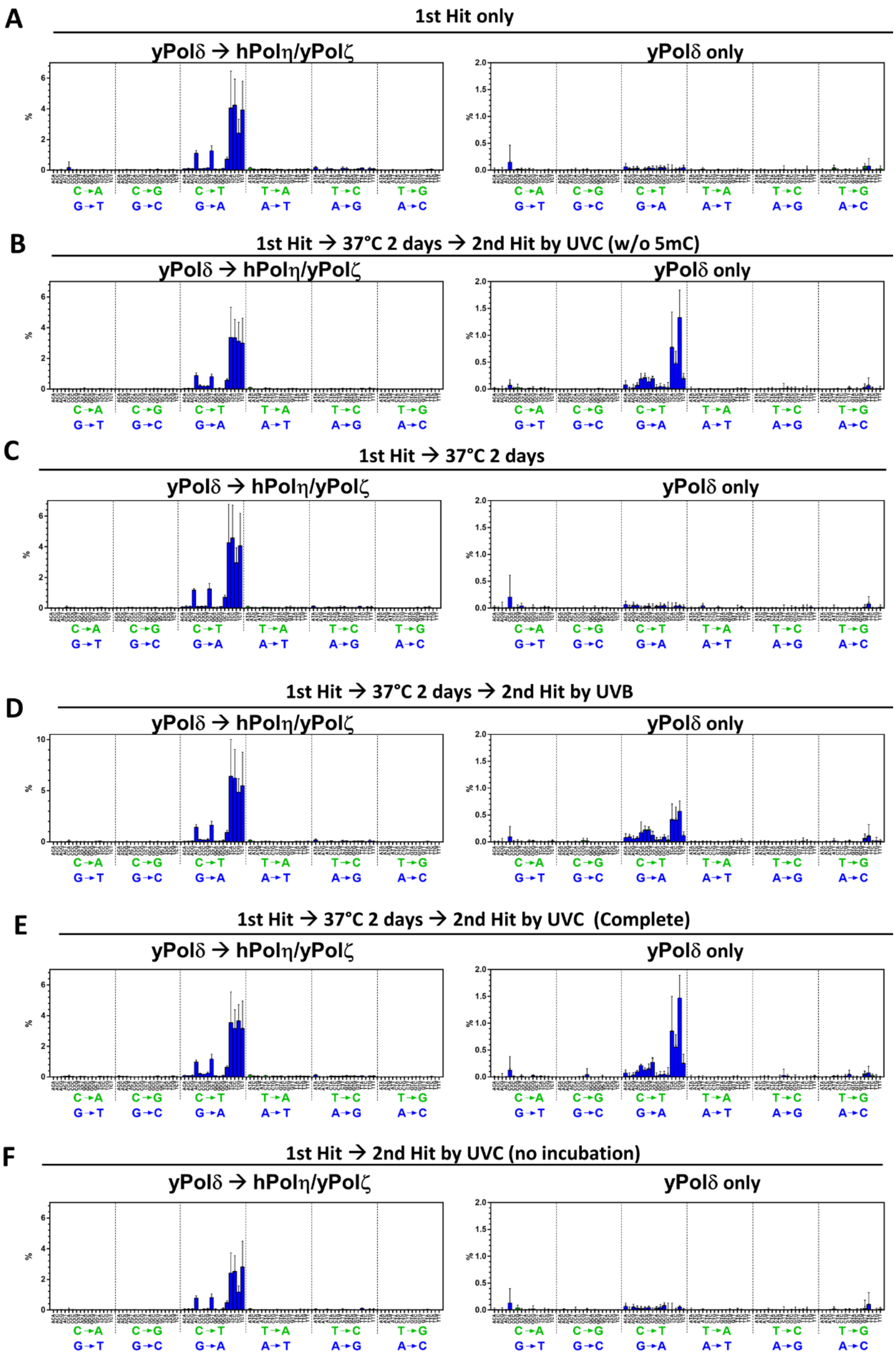

Supplementary Figure S4. Template dsDNA (template A-G) were processed as illustrated in **Figure 4A** (panel **E,** “complete”) or partially processed as indicated, and used for the primer extension only by yPol δ (“yPolδ only”), or by yPol δ first and then followed by premixed of hPol η and yPol ζ (“yPolδ→hPolη/yPolζ”). Products were analyzed by NGS and UV-induced nucleotide substitutions spectra were obtained from frequencies of single nucleotide misincorporations.

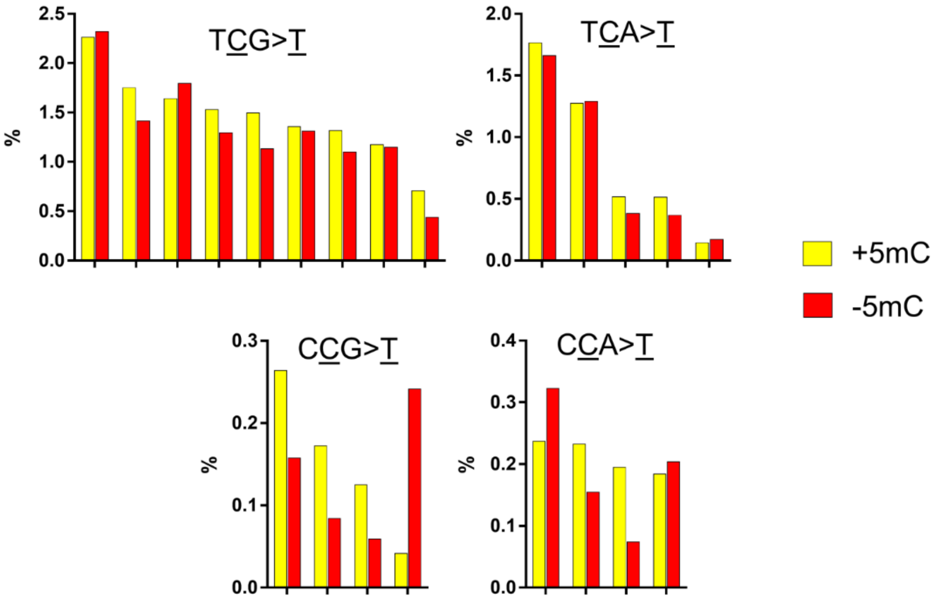

Supplementary Figure S5. Influence of 5mC in deamination-mediated C>T transition. Frequencies of C>T transition (A-misincorporation at template C residue) were analyzed at individual template sites that had indicated trinucleotide contexts. Paired yellow and red bars are C>T transition frequencies at the same sites on the templates that were treated by the complete two-hit deamination procedure as illustrated in **Figure 4A** with (yellow bars) and without (red bars) the methyltransferase treatment (data in **Figure 4K, and E**). If there is an impact by 5mC modification, sites in the left panels should be influenced by the treatment because the dipyrimidines overlap with CpG sequence. Right panels are negative controls, in which dipyrimidines have no overlapping CpG sequence. Although the data may have a trend for 5mC-mediated stimulation of deamination, we concluded it insignificant.

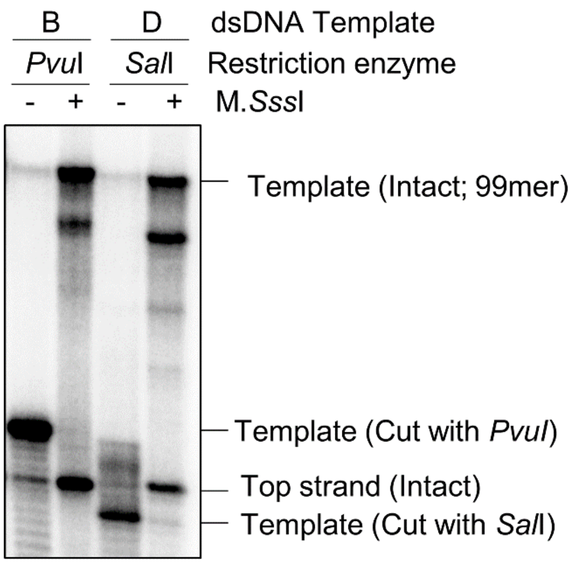

Supplementary Figure S6. Testing methyltransferase modification by restriction digestion. Template B and D have CpG sequence that overlap with *Pvu*I and *Sal*I sites, which are sensitive to the 5mC modification. Indicated dsDNA templates (0.1 pmol) that were treated (+) or untreated (-) with M.*Sss*I methyltransferase were end-labeled with ^{32}P by T4 polynucleotide kinase, and then incubated with indicated restriction enzymes (3 units) for 1 hour at 37°C in the buffers that were supplied from manufacturer. Then the products were separated with a DNA sequencing gel electrophoresis and visualized by a BioRad personal FX phosphorimager.

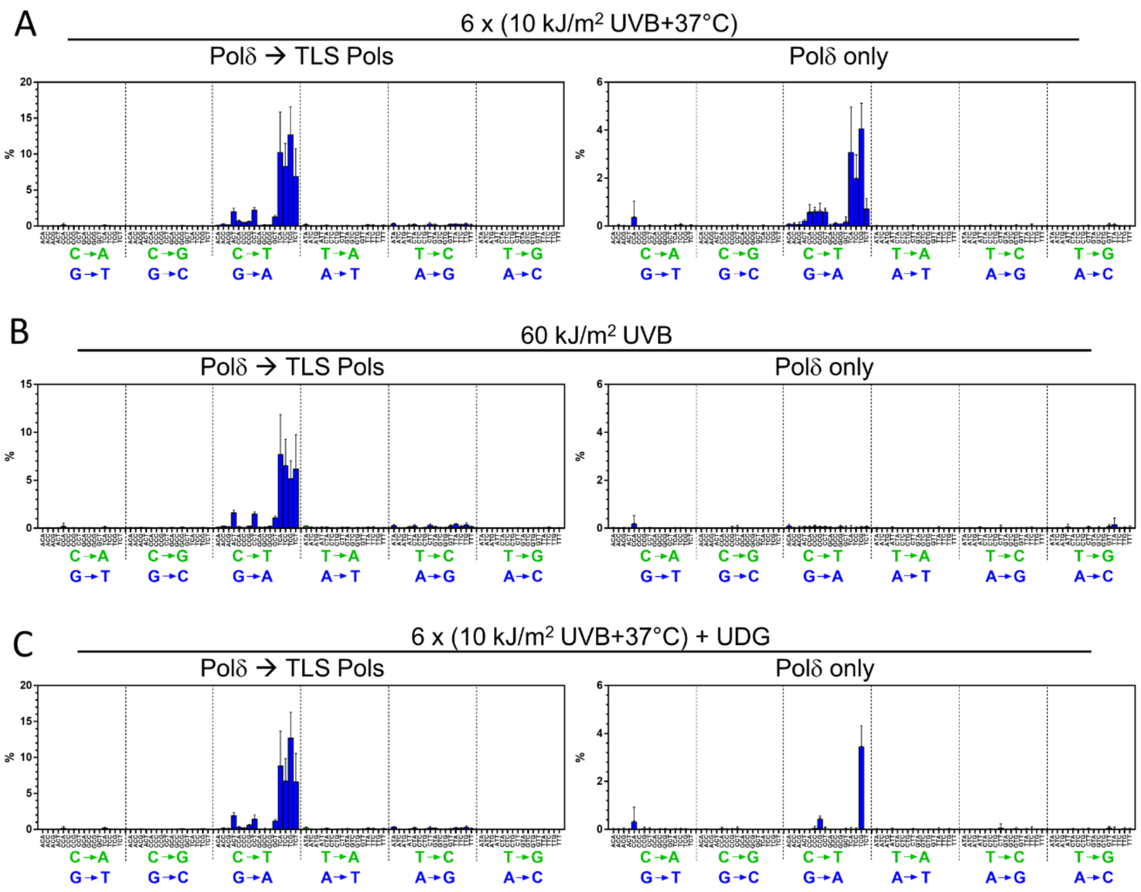

Supplementary Figure S7. Methylated dsDNA templates (template A-G) were processed as illustrated in **Figure 6A** (**A**), single irradiation with 60 kJ/m^2 of UVB (**B**), or processed as in panel-A and then treated by UDG (**C**). The templates were used for the primer extension first by yPol δ and then by premixed hPol η and yPol ζ (Pol δ +TLS Pols), or only by yPol δ. Products were analyzed by NGS and UV-induced nucleotide substitutions spectra were obtained from frequencies of single nucleotide misincorporations.

References

1. Johnson, R.E., Washington, M.T., Haracska, L., Prakash, S. and Prakash, L. (2000) Eukaryotic polymerases iota and zeta act sequentially to bypass DNA lesions. *Nature*, 406, 1015-1019.
2. Tissier, A., McDonald, J.P., Frank, E.G. and Woodgate, R. (2000) poliota, a remarkably error-prone human DNA polymerase. *Genes Dev*, 14, 1642-1650.
3. Vaisman, A., Tissier, A., Frank, E.G., Goodman, M.F. and Woodgate, R. (2001) Human DNA polymerase iota promiscuous mismatch extension. *J Biol Chem*, 276, 30615-30622.
4. Zhang, Y., Yuan, F., Wu, X. and Wang, Z. (2000) Preferential incorporation of G opposite template T by the low-fidelity human DNA polymerase iota. *Mol Cell Biol*, 20, 7099-7108.
